# Supplementary material for: Coordinated collective migration and asymmetric cell division in confluent human keratinocytes without wounding
Source: Nat Commun. 2018 Sep 10;9:3665. doi: 10.1038/s41467-018-05578-7 (PMC6131553; doi:10.1038/s41467-018-05578-7)
Supplement: Supplementary file 1 — Supplementary Information [file 41467_2018_5578_MOESM1_ESM.pdf]

Supplementary Figures

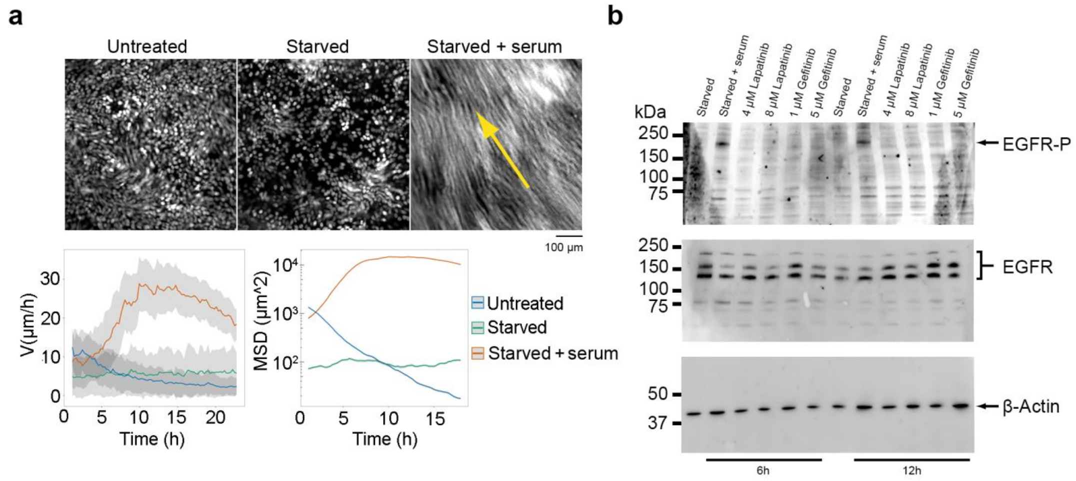

Supplementary Figure 1 Collective migration depends on EGFR signaling. **a** Cell motility in untreated, starved and serum-stimulated cells. Motion is illustrated by cell trajectories formed by projections of 15 time points comprising the period between 9 and 13 h post serum stimulation. Serum was introduced at time point 0. Yellow arrow indicates direction of migration. Images are extracted from Supplementary Movie 2. Graphs show mean velocity ± SD (left panel) and mean square displacement (MSD) (right panel) of cell motions. **b** Western blot showing activation of EGFR in the presence of serum and inactivation in the presence of the EGFR inhibitors gefitinib and lapatinib at the concentrations indicated. Lysates were obtained from cells that had been starved for 48 h and subsequently subjected to serum-stimulation for the time periods indicated.

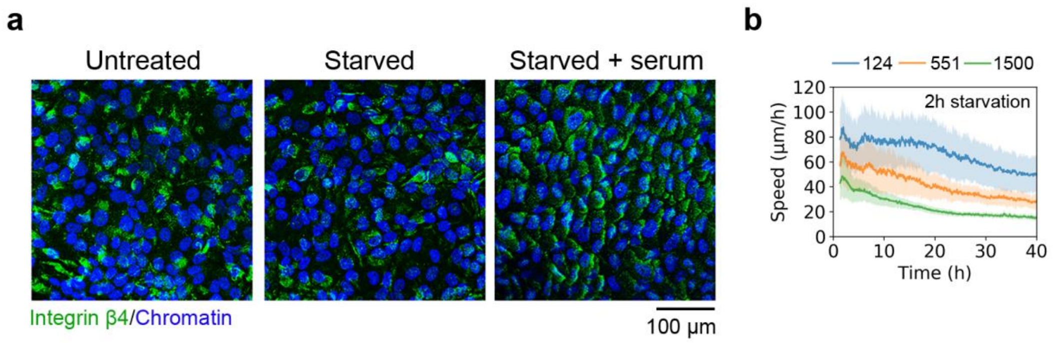

Supplementary Figure 2 a Confocal images illustrating global integrin β4 polarity in collectively migrating cell sheets. Untreated (before starvation), starved (after 48 h starvation but before stimulation) and starved + serum (starved for 48 h and stimulated for 20 h) are shown. **b** Average migration speed generated at different cell densities in the presence of EGF and low calcium concentration. Cells were starved for 2 h prior to stimulation. Mean ± SD are shown; n = 3 experiments.

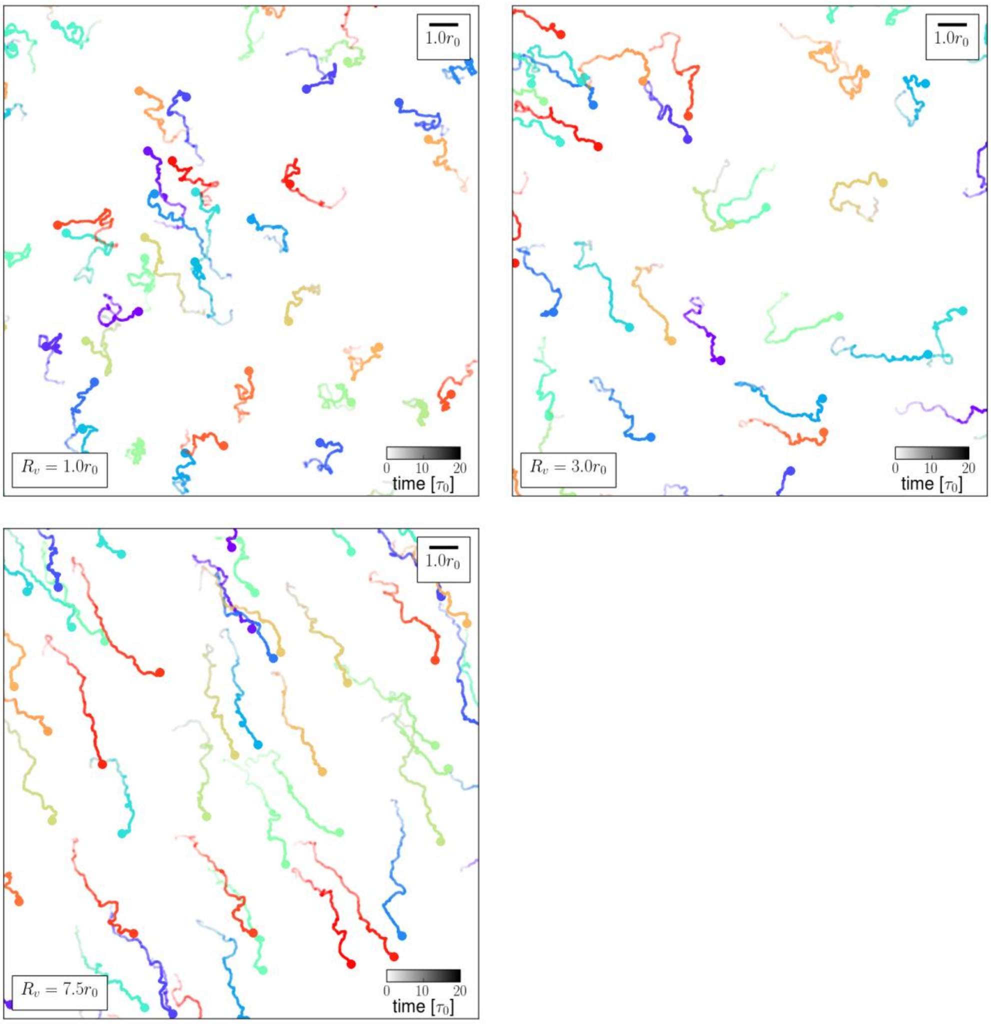

Supplementary Figure 3 Numeric simulations of serum-induced collective migration. Trajectories of active cells obtained from numerical simulations with varying Vicsek radii, visualized over a time period of $t = 20\ \tau_0$.

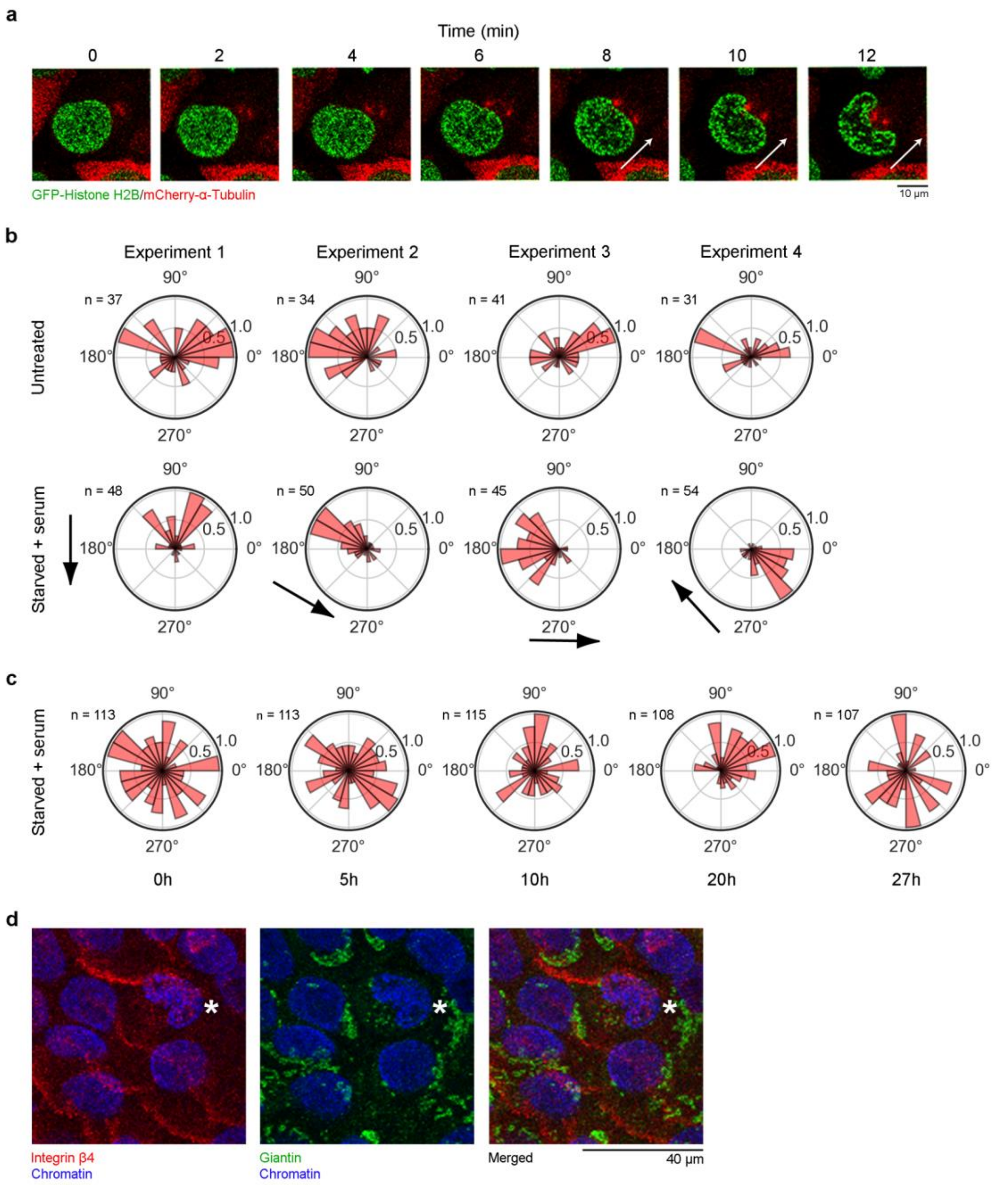

Supplementary Figure 4 Global cell division polarity. In all subfigures HaCaT cells were starved and serum-stimulated for 25 to 30 h prior to analysis, if not indicated otherwise. **a** Time-lapse of HaCaT cells expressing mCherry-α-Tubulin and GFP-Histone H2B. White arrows indicate cell division orientation. **b** Radial diagrams representing the distribution of prophase angle orientations (defined in Fig. 4g) in untreated and serum-stimulated cells are shown for 4

independent experiments. Data were subjected to unity-based normalization (0-1) before plotting. The direction of cell migration is indicated by black arrows. **c** Radial diagrams representing nucleus-to-Golgi alignment in cells serum-stimulated for 0 h, 5 h, 10 h, 20 h and 27 h. The data were subjected to unity-based normalization (0-1) before plotting. Diagrams are representative of single fields of view (OF = 290x290 µm). **d** Reversal of nucleus-to-Golgi configuration at cell division entry. The white asterisk indicates a HaCaT cell in early prophase 27 h after serum stimulation of cells that had been starved for 48 h. Integrin β4 (shown in red) is used as a marker for cell migration orientation.

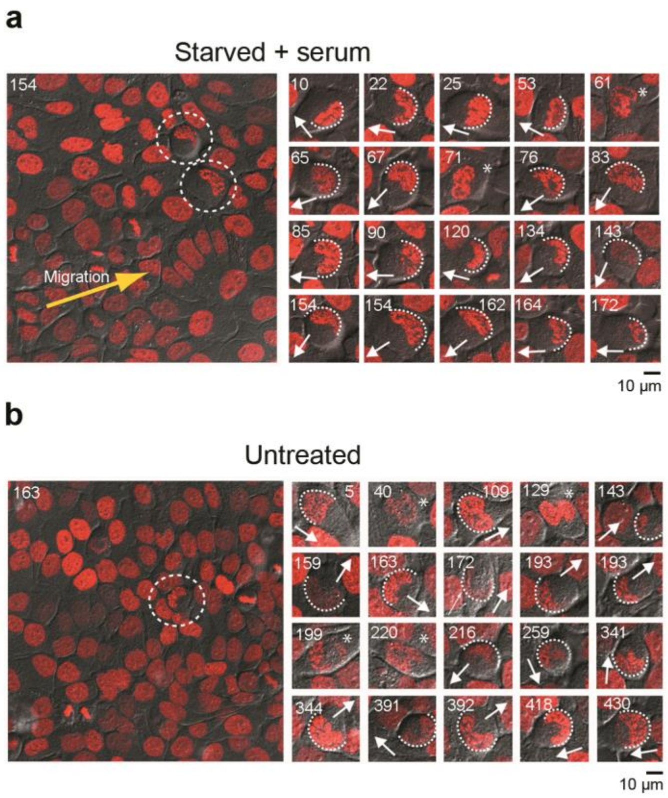

Supplementary Figure 5 Globally oriented cell division polarity in HaCaT cell sheets after starvation and serum stimulation. Monolayers of HaCaT cells stably expressing mCherry-Histone H2B were imaged for 3 to 6 h by confocal live cell microscopy at 1 min intervals between acquisitions. Cells were either **a** grown in the presence of serum-free medium for 2 days followed by re-stimulation in serum-containing medium for 25 h prior to imaging or **b** untreated. Right panels show snapshots of cells at prophase entry organized in chronological order (time points in min are indicated). White arrows indicate prophase orientation. Yellow arrow in the top panel indicates direction of cell migration. Images are extracted from Supplementary Movies 13 and 14.

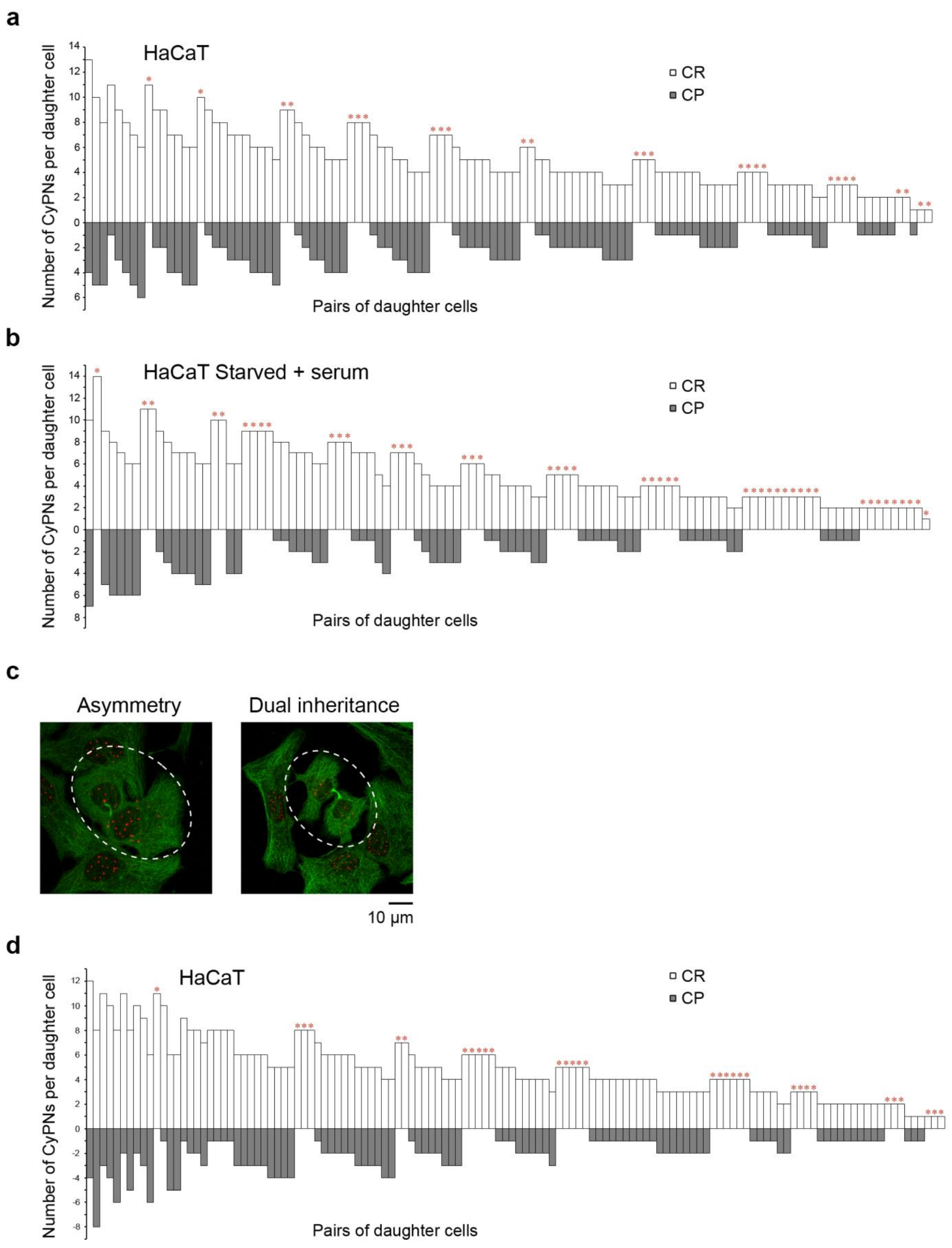

Supplementary Figure 6 Cell pair assays in HaCaT cells. **a** Asynchronously growing HaCaT cells were fixed and labeled using antibodies specific for PML and Aurora B. For each cell pair identified, the number of CyPNs in CR and CP daughter cells were determined and plotted on the top and the bottom of the graph, respectively. Cell pairs with detectable CyPNs in only one of the two daughter cells are defined as complete asymmetric (red asterisk). **b**

HaCaT cells were starved in serum-free medium for 3 days and subsequently released into the cell cycle by adding serum. Cells were fixed 30 h after release from cell cycle block and subsequently processed and analyzed as aforementioned in **a**. **c** Pairs of newly divided daughter cells are identified by antibodies against α-Tubulin (green). Endogenous PML is shown in red. Images represent merged projections of multiple confocal z-sections. White dashed circles highlight a pair of newly divided daughter cells. **d** Cell pair assay using antibodies against α-Tubulin as a cell pair marker. Cells were processed and analyzed as aforementioned in **a**. **a**-**b**, **d** The red asterisk indicates complete asymmetric PML body inheritance. CR, CyPN rich; CP, CyPN poor. The datasets shown are representative of 3 experiments.

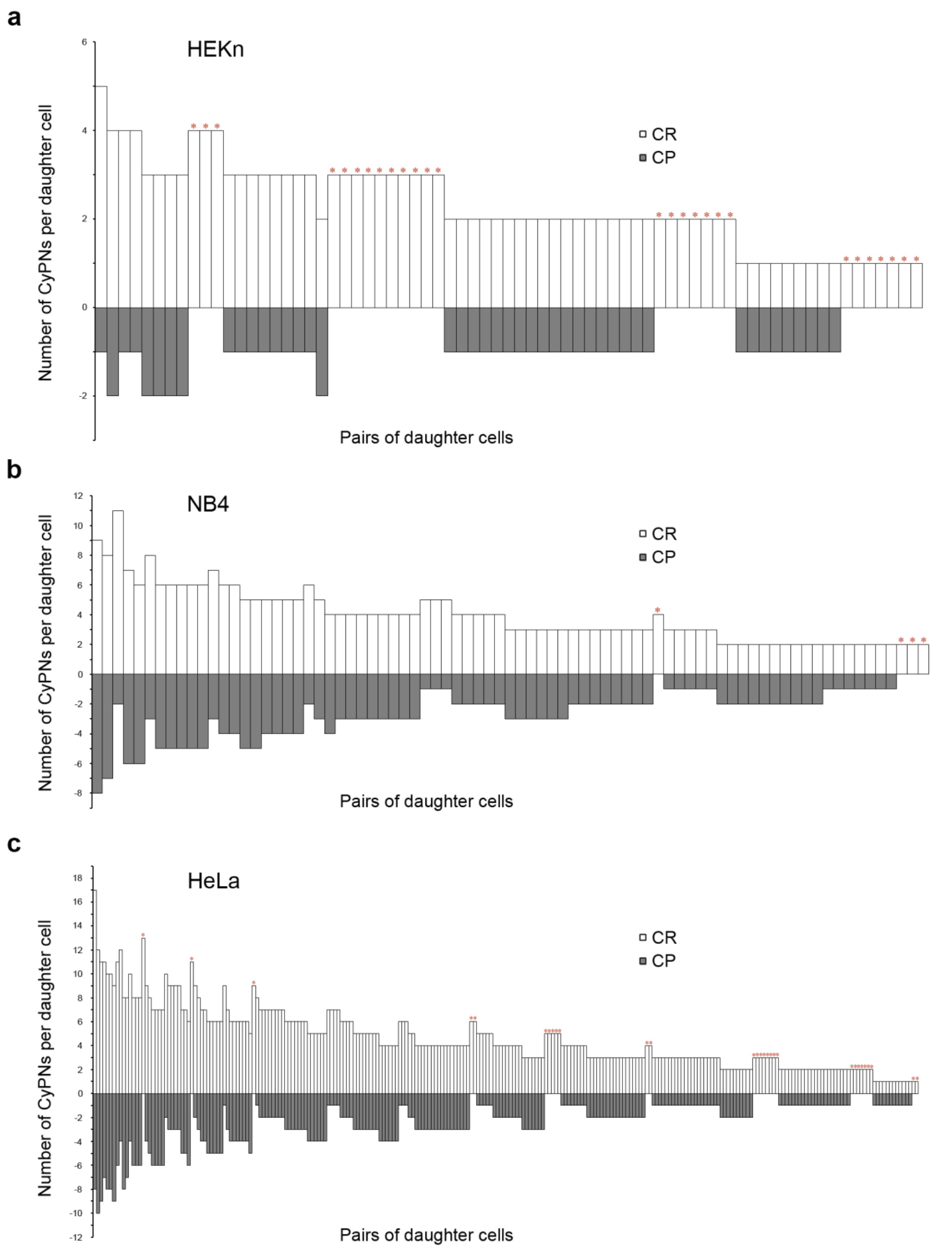

Supplementary Figure 7 Cell pair assay in HEKn, NB4 and HeLa cells. Ratio of asymmetric segregation of PML bodies in **a** human primary keratinocytes (HEKn), **b** NB4 and **c** HeLa cells. **a-c** The red asterisk indicates complete asymmetric PML body inheritance. CR, CyPN rich; CP, CyPN poor. The datasets shown are representative of 3 experiments.

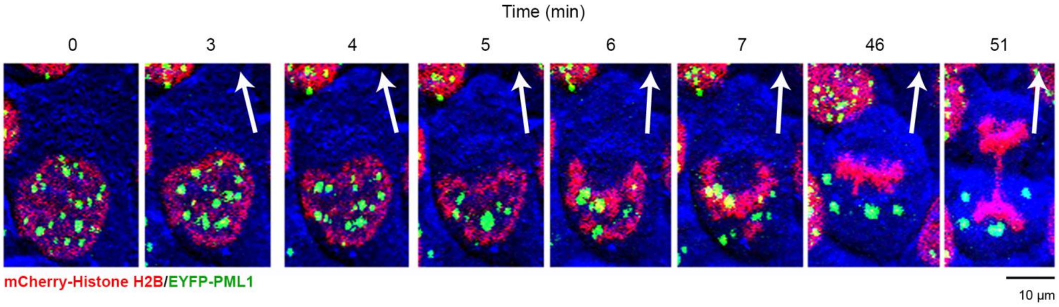

Supplementary Figure 8 PML bodies segregate non-randomly along a mitotic polarity axis. Time-lapse of a HaCaT cell exhibiting complete asymmetric partitioning of PML bodies. EYFP-PML1 (green), mCherry-Histone H2B (red) and DIC (blue) is shown. Arrows indicate the cell division orientation.

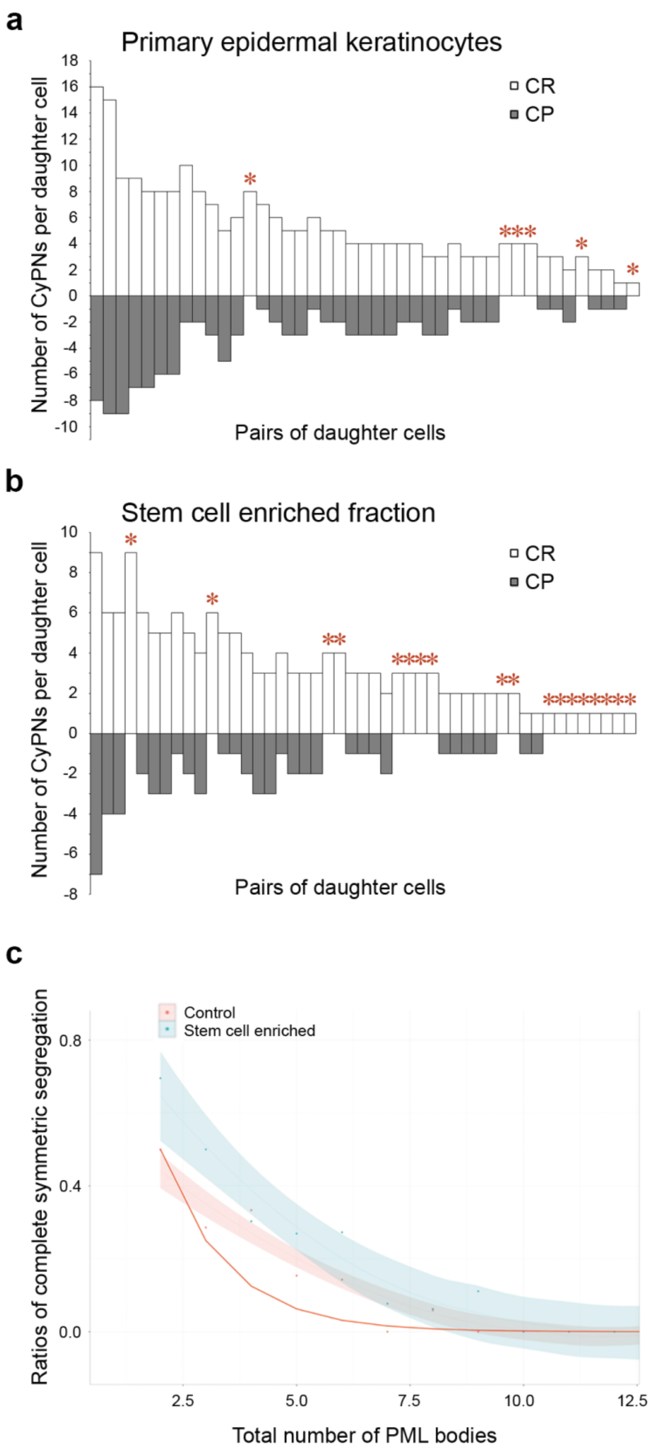

Supplementary Figure 9 Cell pair assay in human primary epidermal keratinocytes. Ratio of asymmetric segregation of PML bodies in **a** human primary epidermal keratinocytes and **b** a stem cell enriched fraction. **a–b** The red asterisk indicates complete asymmetric PML body inheritance. CR, CyPN

rich; CP, CyPN poor. The datasets shown are representative of 3 experiments. **c** Incidence of cell divisions showing complete asymmetric distribution in primary epidermal keratinocytes. Smoothed trend lines are shown as dashed lines with a 95% CI. The theoretically predicted curve for a completely symmetric distribution (p_{sym}=0.5) is shown for comparison as a solid red line. Control (n = 135); Stem cell enriched (n = 231).

Supplementary Information Figure 10

In a previous study the Snap-tag labeling system was used in order to distinguish between aged and new mitochondria[1]. In these experiments an immortalized mammary epithelial cell line was stably transduced by a lentivirus expressing a Snap-tagged version of the mitochondrial outer membrane protein OMP25. By consecutive labeling of the Snap-tagged protein with two different fluorophores they demonstrated asymmetric inheritance of aged but not new mitochondria. Interestingly, the authors of this paper were capable of separating cells containing old and new mitochondria by using fluorescence activated cell sorting (FACS). We were interested in repeating this experiment in HaCaT cells because 1) we were interested in knowing if aged mitochondria co-segregate with PML bodies during cell division and 2) we wanted to separate HaCaT cells with different cell fate. We established HaCaT cells that stably express EGFP-Histone H2B in combination with Snap-tagged OMP25 (expressed from the same lentivirus construct used in the paper by Katajisto *et al.*)[1]. By consecutive labeling of these cells using the Snap substrates TMR-Star (which we presume represent the red label in the paper by Katajisto *et al.*) as the label for new mitochondria

and 647-SiR as the label for aged mitochondria, we found that the TMR-Star and not 647-SiR become asymmetrically apportioned after cell division. By performing additional control experiments, we found that 1) the TMR-Star label localizes to regions that are more perinuclear compared to the 647-SiR staining irrespective of whether it was used as a young or old label, and 2) TMR-Star cross-reacts with cytoplasmic vesicles in cells that do not express a Snap-tagged protein. Thus, in HaCaT cells, differential localization and asymmetric apportioning of the TMR-Star label does not depend on mitochondrial aging. Rather it appears to cross-react with cytoplasmic organelles distinct from mitochondria. To determine the identity of these organelles we co-stained HaCaT cells with TMR-Star and the lysosome/late endosome marker Lysotracker Green (LTG). TMR-Star and LTG showed high degree of co-localization indicating that TMR-Star cross-reacts with lysosomes and that lysosomes are asymmetrically segregated in dividing HaCaT cells.

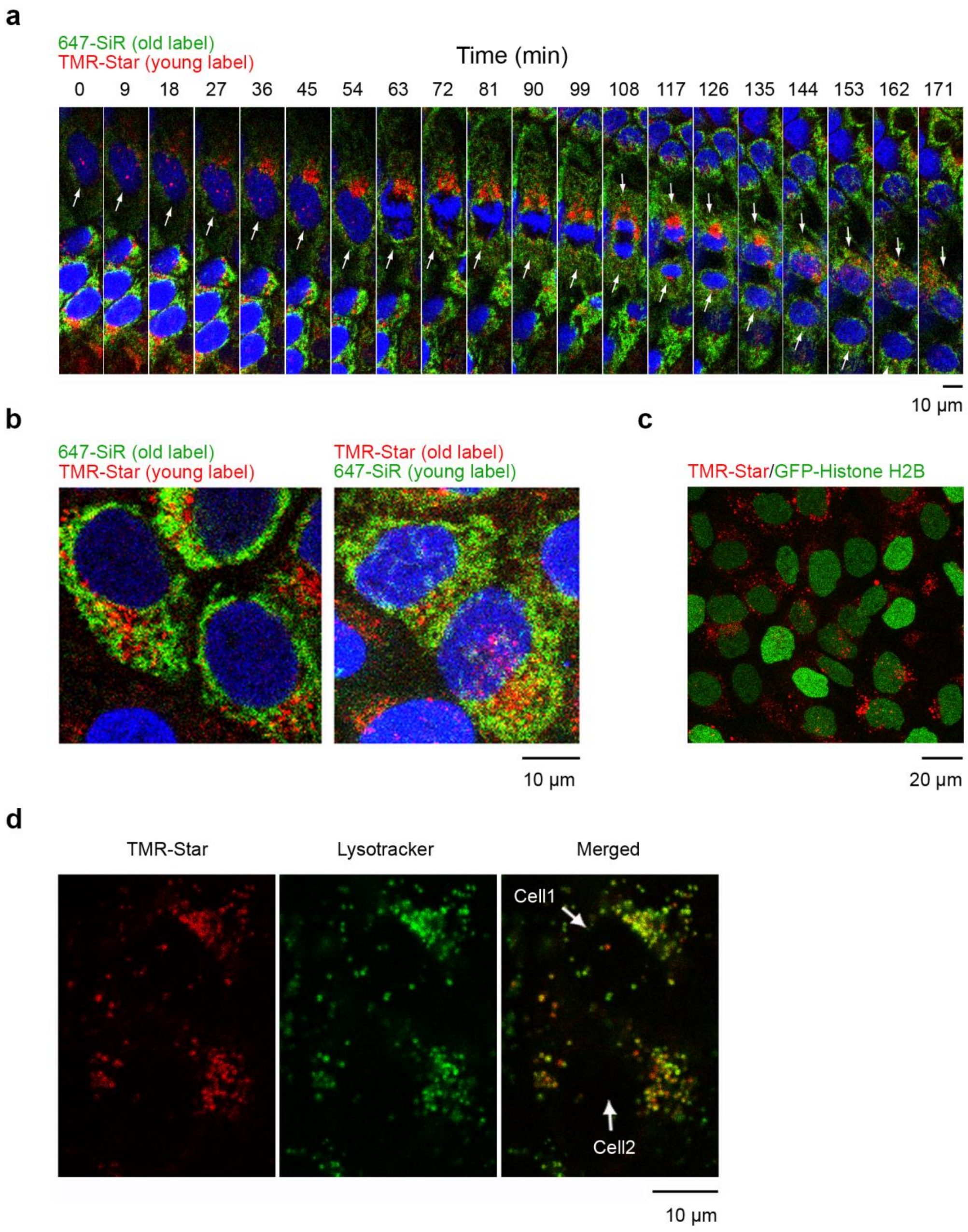

Supplementary Figure 10 The Snap-tag substrate TMR-Star reacts with lysosomes. **a** HaCaT cells stably expressing EGFP-Histone H2B (blue) and Snap-tagged OMP25 were labeled by 647-SiR (green) 30 h prior to imaging and TMR-Star (red) 3 h prior to imaging. Confocal z-stacks were acquired at 3 min intervals. White arrow marks a dividing cell. **b** Live HaCaT cells stably expressing EGFP-Histone H2B (blue) were labeled with 647-SiR for 30 h and TMR-Star for 3 h (left panel) and TMR-Star for 30 h and 647-SiR for 3 h (right

panel). TMR-Star and 647-SiR are visualized in red and green, respectively. Projections of multiple stacks are shown. **c** Live HaCaT cells expressing EGFP-Histone H2B (green) were labeled with TMR-Star in the absence of a Snap-tagged transgene. Cells were imaged 3 h after labeling. TMR-Star reacts with cytoplasmic vesicles. **d** TMR-Star positive vesicles co-localize with lysosome/late endosomes. Live HaCaT cells were labeled with TMR-Star and LTG and imaged 3 h later.

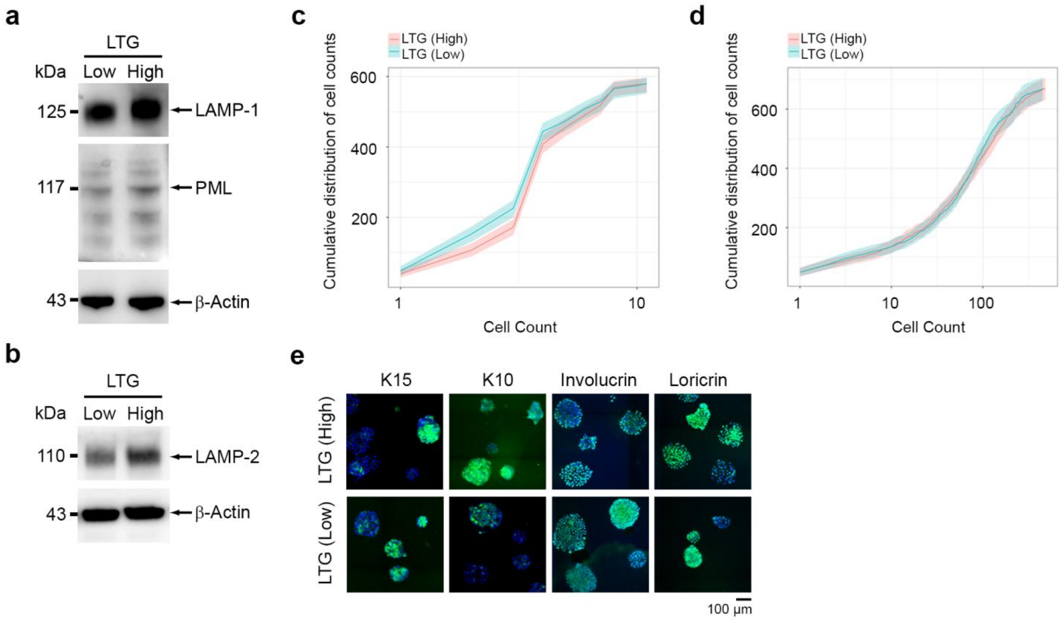

Supplementary Figure 11 Characteristics of LTG sorted cells. Western blot analysis of **a** PML and LAMP-1 and **b** LAMP-2 in LTG(High) and LTG(Low) cells 2 days after plating. The molecular sizes (kDa) are indicated. β-Actin was used as loading control. **c** Cumulative distribution of cell counts in LTG(High) and LTG(Low) cell colonies 2 days after plating. LTG(High) (n = 608); LTG(Low) (n = 614). 3 experiments were performed. **d** Cumulative distribution of cell counts in LTG(High) and LTG(Low) cell colonies 6 days after plating. LTG(High) (n = 695); LTG(Low) (n = 816). 3 experiments were performed. **e** IF analysis of LTG(High) and LTG(Low) cell colonies 6 days after plating using antibodies specific for K10, K15, Involucrin and Loricrin (green). DAPI is shown in blue. Representative images are shown; n = 5.

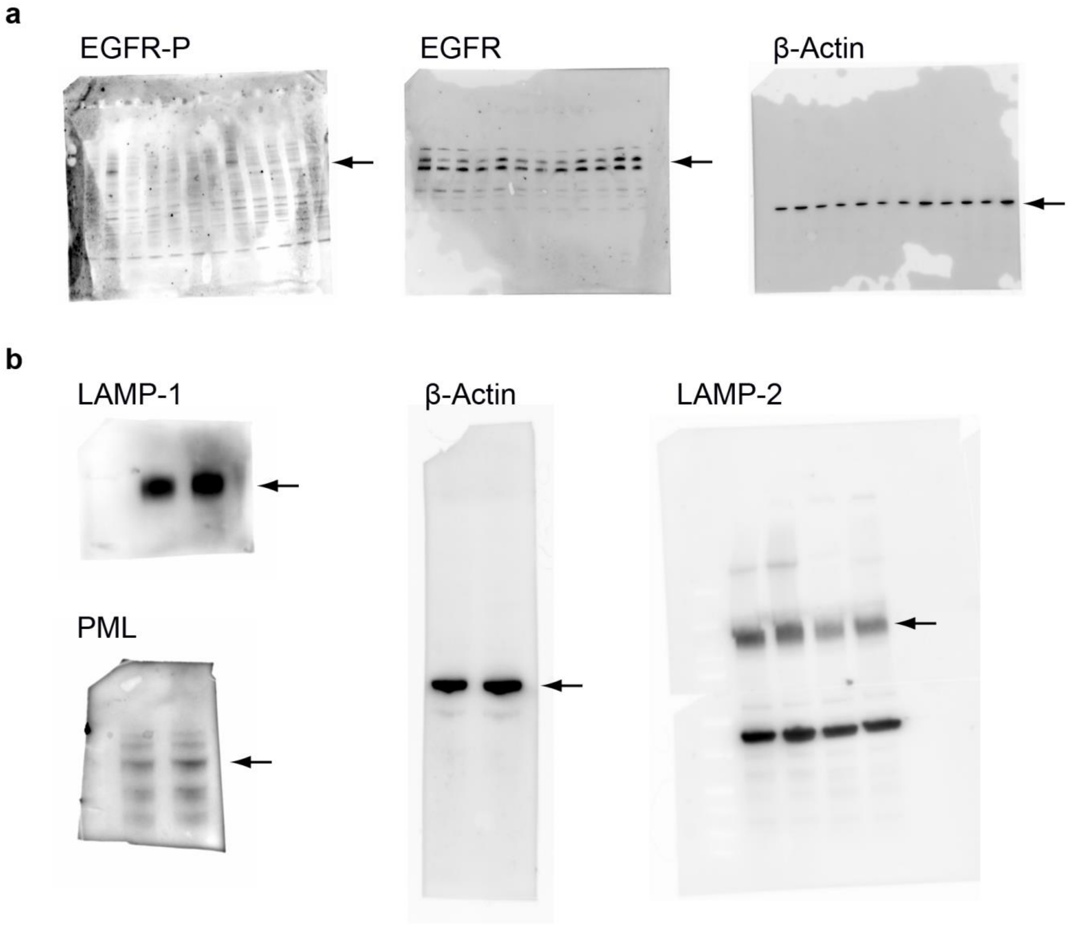

Supplementary Figure 12 Original blots from Western analysis. **a** Uncropped scans of blots shown in Supplementary Fig. 1. **b** Uncropped scans of blots shown in Supplementary Fig. 11. Black arrows point to specific protein bands.

Supplementary Methods

Antibodies, fluorescent reagents and drugs

Primary antibodies: mouse anti-PML (dilution 1:400; sc-966; Santa Cruz Biotechnology), rabbit anti-PML (dilutions IF 1:400, WB 1:10000; NB100-59787; Novus Biologicals), rabbit anti-Aurora B (dilution 1:500; ab2254; Abcam), mouse anti-α-Tubulin (dilution 1:100; T6199; Sigma-Aldrich), mouse anti-LAMP-1 (dilutions IF 1:200, WB 1:1000; H4A3; Santa Cruz Biotechnology), mouse anti-LAMP-2 (dilution 1:500; H4B4; Abcam), rabbit

anti-Giantin (dilution 1:1000; Poly19243; Covance), mouse anti-Integrin β4 (dilution 1:500; MAb1964; Millipore), mouse anti-Integrin β4 (dilution 1:500; MAB2059Z; Millipore), rat anti-CD49f (dilution 1:400; 313614; BioLegend), rabbit anti-Cytokeratin 15 (dilution 1:200; EPR1614Y; Abcam), mouse anti-Cytokeratin 10 (dilution 1:200; RKSE60; Abcam), rabbit anti-Involucrin (dilution 1:100; ab53112; Abcam), rabbit anti-Loricrin (dilution 1:100; ab24722; Abcam), rabbit anti-EGFR (dilution 1:1000; ab32077; Abcam), rabbit anti-EGFR-P (dilution 1:1000; ab40815; Abcam), mouse anti-β-Actin (dilution 1:30000; AC-15; Santa Cruz Biotechnology) and rabbit anti-Histone H3 (dilution 1:800; ab5176; Abcam). Secondary antibodies: Alexa Fluor® 647 rat anti-human CD49f (dilution 1:400; 562494; BD Biosciences), Alexa Fluor® 488 goat anti-mouse IgG (dilution 1:400; A-11029; Life Technologies), Alexa Fluor® 488 goat anti-rabbit IgG (dilution 1:400; A-11034; Life Technologies) and Alexa Fluor® 594 goat anti-rabbit IgG (dilution 1:400; A-11012; Life Technologies). For western blotting we used alkaline phosphatase-conjugated anti-rabbit and anti-mouse antibodies from Santa Cruz Biotechnology (dilution 1:10000; sc-2007 and sc-2047, respectively). Fluorescent reagents: Alexa Fluor® 594-conjugated Phalloidin (dilution 1:1000; A12381; Thermo Fisher Scientific), CytoPainter Phalloidin-iFluor 488 Reagent (dilution 1:1000; ab176753; Abcam), LysoTracker® Green DND-26 (60nM; L7526; Thermo Fisher Scientific), SNAP-Cell® 647-SiR (2µM; S9102S; New England BioLabs) and SNAP-Cell® TMR-Star (2µM; S9105S; New England BioLabs). Drugs: Lapatinib (4µM; CDS022971; Sigma-Aldrich), Gefitinib (1µM; Y0001813; Sigma-Aldrich), Vacuolin-1 (1µM; #673000; Millipore), Glycyl-L-

phenylalanine 2-naphthylamide (0.2mM; sc-252858; Santa Cruz Biotechnology), Nocodazole (100 ng/ml; M1404; Sigma-Aldrich).

Lentiviruses

The lentiviral plasmid pLNGY-PML.I expressing EYFP-tagged PML1 was obtained from Roger Everett at University of Glasgow[2]. Lentiviral plasmids expressing mCherry-Histone H2B or GFP-Histone H2B were obtained from Addgene[3]. Lentiviral plasmid expressing mCherry-α-Tubulin (pCDH-EF1a_mCherry–α-Tubulin_Bsd) was obtained from Coen Kampstejn at Oslo University Hospital[4]. Lentivirus plasmid expressing Snap-OMP25 was obtained from Addgene (#69599)[1]. Production of lentiviral particles and establishment of stably transduced HaCaT cells was performed as previously described[5].

Immunofluoresence (IF) analysis

Cells were fixed and processed for IF analysis as previously described[6]. The primary epidermal cells were grown on Thermanox plastic coverslips, while all other cell lines were grown on glass coverslips. In brief, adherent cells were fixed in 100% methanol (VWR) for 5 min or 4% paraformaldehyde (PFA; Sigma-Aldrich) in PBS for 10 min, followed by permeabilization in 0.25% Triton X-100 (Sigma-Aldrich) for 4 min. Suspension cells (NB4) were mounted on coverslips using cytospin. After incubation in blocking buffer (1x PBS, 0.5% BSA) for 15 min, primary antibody staining was performed at 4 °C ON, followed by secondary antibody staining at 37 °C for 2 h. Cells were mounted in Vectashield containing DAPI (Vector Laboratories). Cells grown on plastic

coverslips were inverted on MatTek glass bottom dishes (MatTek Corporation) prior to imaging. Images were acquired using a Leica TCS SP8 confocal microscope equipped with a 40x 1.30 NA oil immersion lens and a Zeiss wide field Axiobserver Z1 microscope equipped with a 10x 0.3 NA air objective.

Protein extraction and western blotting

Total protein extracts were prepared by washing cells twice in PBS and subsequently lyse cells in urea extraction buffer containing 8 M urea (Duchefa Biochemie), 1% Triton X-100 (Sigma-Aldrich) and 10 mM dithiothreitol (DTT; Sigma-Aldrich), followed by cell rupture using QIAshredder (QiAgen). Protein samples were run on 10% NuPAGE Bis-Tris gels (Life Technologies), transferred to Immobilon nylon filter (Millipore) and subsequently probed with primary and secondary antibodies. Uncropped western blots are shown in Supplementary Fig. 12.

Quantification of cell division by flow cytometry

HaCaT cells seeded in 6 cm dishes were starved for 2 days and subjected to serum stimulation. Cells harvested at different time points after stimulation were washed twice with PBS and fixed in ice-cold 70% ethanol. Cells were incubated in the presence of antibodies against phosphorylated Histone H3 for 1 h at RT, washed with PBS and incubated with secondary antibody Alexa 488 goat anti-rabbit IgG for 30 min at RT. Finally, cells were resuspended in PBS containing 10 µg/ml RNase A (Qiagen) and 20 µg/ml propidium iodide

(Sigma-Aldrich). Samples were analyzed by flow cytometry using Accuri C6 (BD Biosciences).

Analysis of cell cycle length and 2-cell colony rotation

Sorted LTG(High) and LTG(Low) HaCaT keratinocytes were seeded at clonal densities in 12-well glass bottom dishes from MatTek. Cells were then placed in a CO2 incubator at 37 °C for 6 h before they were moved to the microscope incubation chamber. Following a 1 h equilibration period, imaging was performed using a 20x 0.8 NA air objective attached to a wide field microscope. A tailed grid consisting of 16 images were acquired per time point at a frame rate of 4 min between acquisitions. Only adherent cells that were not disturbed by floating cells or debris were analyzed.

Colony size analysis

Sorted LTG(High) and LTG(Low) HaCaT keratinocytes were plated at clonal densities in 6-well plastic dishes. At day 2 and 6 after plating cells were fixed in 4% PFA (Sigma-Aldrich) and subsequently permeabilized using 0.4% Triton X-100 (Sigma-Aldrich). Nuclei were then labeled using DAPI. Images were made using a 10x 0.45 NA air objective attached to a wide field microscope. A tailed grid consisting of 10 images were acquired and the number of cells in each colony was determined using the ImageJ plugin Particle analysis.

Expression of stem cell and differentiation markers

LTG(Low) and LTG(High) cell colonies were subjected to IF 6 days after plating, using antibodies against K10, K15, Involucrin and Loricrin. Images

were acquired with a 10x air objective attached to a wide field microscope. Each image represents a stitched grid of 25 (5x5) fields of view. All colonies with specific antibody staining were determined as positive regardless if the whole colony was positive or just a few cells.

Statistical analysis

Data are expressed as mean ± standard deviation (SD) (Fig. 1b-c, 1e-g; 2a-b, 2d; 5c, 5h; 7e, 7g-h; Supplementary Fig. 1a; 2b), mean ± standard error of mean (SEM) (Fig. 4f), median ± min to max value (Fig. 4i-j) or median ± 5-95 percentiles (Fig. 7c-d), quantified from independent experiments. Error bars indicate the differences, which were considered significant when p was less than 0.05 (ns, $p > 0.05$; *, $p \leq 0.05$; **, $p \leq 0.01$; ***, $p \leq 0.001$; ****, $p \leq 0.0001$). Statistical significance was calculated using unpaired Student's t test (Fig. 5c, 5h; 6f; 7c-e, 7g-h) or non-parametric Wilcoxon rank sum test unpaired (Fig. 4i) and paired (Fig. 6b, 6d) using Microsoft Excel, GraphPad and R software. For calculation of mean cell division vector magnitude (Fig. 4i), we consider a single cell division event as a process occurring along a unit vector with an orientation defined as illustrated in Fig. 4g. The mean of these unit vectors will have an average direction defining the dominant orientation of cell divisions in the sample, and a magnitude between zero and one which defines the proportion of cell division vector components in the direction of the dominant orientation. A sample containing cells which all divide in a consistent orientation would therefore have a magnitude of 1 in the dominant direction, while a large enough sample of dividing cells with purely random orientation would have a random dominant axis and a magnitude

tending to zero. We used these magnitudes to compare the treatment-dependent tendency of cells to divide along a consistent by performing a non-parametric Wilcoxon rank sum test, which returns a p-value of 0.02857, implying statistical significance for this observation. As a confirmation that this p-value is not achieved serendipitously, we have also performed a Student's t-test on 1000 resampled replicates of these group magnitudes and found the median p-value to be 0.0195, with 78% of all of these p-values lying below 0.05. Each of the four experiments presented in Fig. 4i and Supplementary Fig. 4b consists of between 31 and 54 orientation measurements. Using bootstrap resampling (500 replicates) we estimate 90% confidence intervals (CI) for each set of vector magnitudes and find that the upper extent of the 90% CI is always less than 0.138 above the measured mean for untreated cells, while the lower extent of the 90% CI for starved and serum-stimulated cells is always less than 0.142 below the relevant mean. In practice this means that there is only one untreated sample which overlaps with the 90% CI of the starved and serum-stimulated cells, suggesting that this point could be reasonably classified as an outlier. The distribution of PML bodies between daughter cells (Fig. 5d; Supplementary Fig. 9c) was modeled as a set of Bernoulli trials, where PML bodies are assigned to one daughter cell with a probability p, or the other daughter cell with a probability of 1-p. The distribution of PML bodies between daughter cells follows a binomial distribution, with a finite probability in each case that one daughter cell will not inherit any PML bodies. Average PML distributions which are totally symmetrical are generated when p=0.5. Deviation of p from 0.5 will result in increasing degrees of asymmetry in the distribution of PML bodies between

daughter cells. We calculated the expected proportion of empty daughter cells for any number of PML bodies to find the assignment probability p which most closely represents our observations, using a non-linear least-squares fitting method.

Supplementary References

1. Katajisto, P. *et al.* Stem cells. Asymmetric apportioning of aged mitochondria between daughter cells is required for stemness. *Science (New York, N.Y.)* **348**, 340-343, doi:10.1126/science.1260384 (2015).
2. Cuchet, D. *et al.* PML isoforms I and II participate in PML-dependent restriction of HSV-1 replication. *Journal of cell science* **124**, 280-291, doi:10.1242/jcs.075390 (2011).
3. Kita-Matsuo, H. *et al.* Lentiviral vectors and protocols for creation of stable hESC lines for fluorescent tracking and drug resistance selection of cardiomyocytes. *PloS one* **4**, e5046, doi:10.1371/journal.pone.0005046 (2009).
4. Vietri, M. *et al.* Spastin and ESCRT-III coordinate mitotic spindle disassembly and nuclear envelope sealing. *Nature* **522**, 231-235, doi:10.1038/nature14408 (2015).
5. Lång, E. *et al.* The arsenic-based cure of acute promyelocytic leukemia promotes cytoplasmic sequestration of PML and PML/RARA through inhibition of PML body recycling. *Blood* **120**, 847-857, doi:10.1182/blood-2011-10-388496 (2012).
6. Jul-Larsen, A. *et al.* PML-nuclear bodies accumulate DNA in response to polyomavirus BK and simian virus 40 replication. *Experimental cell research* **298**, 58-73, doi:10.1016/j.yexcr.2004.03.045 (2004).
