# Supplementary material for: Coordinated collective migration and asymmetric cell division in confluent human keratinocytes without wounding
Source: Nat Commun. 2018 Sep 10;9:3665. doi: 10.1038/s41467-018-05578-7 (PMC6131553; doi:10.1038/s41467-018-05578-7)
Supplement: Supplementary file 3 — Description of Additional Supplementary Files [file 41467_2018_5578_MOESM3_ESM.pdf]

Description of Additional Supplementary Files

File Name: Supplementary Movie 1
Description: Long-range collective migration following serum re-stimulation of starved cells. HaCaT cells stably expressing mCherry-Histone H2B were starved for 2 days prior to serum stimulation. The time-lapse video was acquired using a 20x air objective attached to a wide field microscope at 4 min between frames (the video shown has been reduced to 96 min between frames). For each time point, a grid consisting of 100 (10x10) fields of view were acquired and stitched. Image acquisition started 1 h after serum stimulation. The movie comprises 38.4 h of real time imaging. Scale bar, 1 mm.

File Name: Supplementary Movie 2
Description: Collective migration is activated by consecutive serum deprivation and stimulation. Composite of time-lapse movies showing confluent monolayers of HaCaT keratinocytes stably expressing mCherry-Histone H2B. Left panel shows asynchronous cells that have just reached confluence. Middle panel shows cells that have been serum depleted for 2 days. Right panel shows cells that have been serum depleted for 2 days and subsequently re-stimulated with serum. Acquisitions were performed simultaneously in a multiwell plate and started 1 h after serum stimulation. Acquisitions were made at 8 min time intervals (the movie has been reduced to 32 min per frame) and comprise a total of 21.8 h in real time.

File Name: Supplementary Movie 3
Description: Serum-induced collective migration is abrogated by EGFR inhibitors. Composite of time-lapse movies showing quiescent HaCaT/mCherry-Histone H2B keratinocytes stimulated or not with serum in the presence or absence of the EGFR inhibitors lapatinib and gefitinib. Acquisitions of movies were performed simultaneously in a multiwell plate at 8 min time intervals (the movies shown have been reduced to 32 min per frame) and comprise a total of 24.5 h in real time.

File Name: Supplementary Movie 4
Description: Effect of calcium on collective cell migration. Confluent HaCaT/mCherry-Histone H2B cells were subjected to starvation for 48 h and subsequently exposed to CNT-Prime medium containing EGF and indicated concentrations of calcium. Acquisitions of movies were started 1 h after stimulation and were performed simultaneously in a multiwell plate at 8 min time intervals (movies have been reduced to 16 min between frames). Lower panels show particle tracking of mCherry-labeled nuclei.

File Name: Supplementary Movie 5
Description: Effect of cell density in the presence of low calcium. Different densities of HaCaT/mCherry-Histone H2B cells were starved for 48 h and subsequently exposed to CNT-Prime medium containing EGF and 0.07 mM calcium. Acquisitions of movies were performed simultaneously in a multiwell plate at 8 min time intervals (the movies shown have been reduced to 32 min per frame) and comprise a total of 30 h in real time. Indicated cell densities represent average density of multiple wells at time point t = 0 of the acquired movie. Lower panels show particle tracking of mCherry-labeled nuclei.

File Name: Supplementary Movie 6
Description: Live imaging of Alexa647-labeled anti-Integrin α6 in living HaCaT/mCherry-Histone H2B cells. Cells were seeded at low density in a 6 cm glass bottom MatTek glass bottom dish and subsequently subjected to serum deprivation for 48 h. Cells were stimulated with EGF in CNT-Prime medium containing 0.07 mM calcium. At time point t = 20 h Alexa647-conjugated antibodies specific for integrin α6 was added. Labeled cells were subjected to confocal live imaging using a single z-plane at 1 min between frames.

File Name: Supplementary Movie 7
Description: Live imaging of Alexa647-labeled anti-Integrin α6 in collectively migrating HaCaT/mCherry-Histone H2B cells. Confluent cells in a MatTek glass bottom dish were subjected to serum deprivation for 48 h followed by serum stimulation. At time point t = 25 h Alexa647-conjugated antibodies specific for integrin α6 was added. Labeled cells were subjected to confocal live imaging using a single z-plane at 1 min between frames.

File Name: Supplementary Movie 8
Description: Effect of cell density on non-starved cells. Same experiment as for Supplementary Movie 5, except, in this experiment we used a short starvation period of only 2 h instead of 48 h.

File Name: Supplementary Movie 9
Description: Simulated confluent cells in the absence of an active force. The total simulation time shown corresponds to 20 τ_0.

File Name: Supplementary Movie 10
Description: Simulated confluent active cells with a Vicsek radius of $R_V = 7.5\, r_0$. The total simulation time shown corresponds to 20 τ_0.

File Name: Supplementary Movie 11
Description: Simulated confluent active cells with a Vicsek radius of $R_V = 1.0\, r_0$. The total simulation time shown corresponds to 20 τ_0.

File Name: Supplementary Movie 12
Description: Forward nuclear migration prior to mitosis in HaCaT cells. Time-lapse of HaCaT keratinocytes stably expressing mCherry-Histone H2B (red) in combination with DIC (gray) were used to track the position of the nucleus in relation to the plasma membrane prior to mitosis. Images were captured after 48 h starvation and 25 h of serum stimulation at 1 min time intervals between frames.

File Name: Supplementary Movie 13
Description: Globally oriented cell division polarity in HaCaT cell sheets after starvation and serum stimulation. HaCaT cells stably expressing mCherry-Histone H2B were grown in the presence of serum-free medium for 2 days followed by re-stimulation in serum-containing medium for 25 h prior to imaging. Time-lapse of the cells were imaged for 6 h by confocal live cell microscopy at 1 min intervals between acquisitions. Projections of multiple z-scans are shown. White arrows indicate prophase orientation.

File Name: Supplementary Movie 14
Description: Cell division polarity in non-starved HaCaT cells. Time-lapse of asynchronously HaCaT keratinocytes stably expressing mCherry-Histone H2B were imaged for 6 h by confocal live cell microscopy at 1 min intervals between acquisitions. Projections of multiple z-scans are shown. White arrows indicate prophase orientation.

File Name: Supplementary Movie 15
Description: Polarized mitosis and asymmetric inheritance of PML bodies. Time-lapse of HaCaT keratinocytes expressing EYFP-PML1 (green) and mCherry-Histone H2B (red). The movie is composed of 80 frames that are projections of multiple z-scans. Intervals between frames are 2 min.

File Name: Supplementary Movie 16
Description: TMR-Star co-localizes with lysosomes. Time-lapse of HaCaT keratinocytes labeled with TMR-Star (red) and LTG (green). A single confocal plane shows the cytoplasm of two neighboring cells. The movie is composed of 9 frames, 10 seconds between intervals.

File Name: Supplementary Movie 17
Description: Polarized and asymmetric segregation of TMR-Star. Time-lapse of HaCaT keratinocytes stably expressing GFP-Histone H2B (green) and Snap-OMP25. Cells were starved for 2 days and subsequently serum stimulated. Cells were labeled with 647-SiR (blue) and TMR-Star (red) 5 h prior to and 25 h after serum stimulation, respectively. Time-lapse images were acquired 26 and 31 h post serum stimulation. The movie comprises a total of 86 min, and each time point represents a projection of multiple z-scans. Intervals between frames are 2 min.

File Name: Supplementary Movie 18
Description: Asymmetric segregation of lysosomes. Time-lapse of HaCaT keratinocytes labeled with LTG. Cells were subjected to serum deprivation for 2 days and subsequently serum stimulated. Confocal time-lapse images were acquired between 25 and 30 h after serum stimulation. Projections of multiple z-sections are shown at each time point. Regions of interest used for quantification of LTG intensity in newly divided daughter cells are indicated by yellow rings. The movie comprises a total of 78 min and intervals between frames are 2 min.

File Name: Supplementary Movie 19
Description: Rotational motility of cells seeded at clonal density. HaCaT kerationcytes were sorted into LTG(Low) and LTG(High) cell populations and subsequently seeded on glass bottom dishes at clonal densities. DIC time-lapse series were generated using a 20x air objective attached to a wide field microscope at 4 min between frames (the video shown has been reduced to 16 min between frames). The movie comprises 29.3 h of real time imaging. Representative cell divisions and 2-cell colony rotations for the two populations are shown.

File Name: Supplementary Movie 20
Description: Motility of LTG sorted cells. Composite of time-lapse movies showing differences in motility between LTG(Low) and LTG(High) cells. Sorted cells were seeded on collagen IV-coated glass in the presence or absence of vacuolin-1 or GPN. DIC imaging, starting 6 h after cell plating, was performed using a 20x air objective at 8 min between frames (movies showing were reduced to 32 min per frame). Each frame represents a stitched grid of 16 (4x4) fields of view.
